# Risk factors for the development of cervical cancer: analysis of the evidence

**DOI:** 10.3389/fonc.2024.1378549

**Published:** 2024-05-23

**Authors:** Julissa Luvián-Morales, Sandra Olimpia Gutiérrez-Enríquez, Víctor Granados-García, Kirvis Torres-Poveda

**Affiliations:** ^1^ MICAELA Program, Instituto Nacional de Cancerología, Mexico City, Mexico; ^2^ Faculty of Nursing and Nutrition, Autonomous University of San Luis Potosi, San Luis Potosi, Mexico; ^3^ Epidemiological and Health Services Research Unit Aging Area, Instituto Mexicano del Seguro Social (IMSS), Mexico City, Mexico; ^4^ Center for Research on Infectious Diseases, Instituto Nacional de Salud Pública (INSP), Cuernavaca, Mexico; ^5^ Consejo Nacional de Humanidades Ciencias y Tecnologías (CONAHCYT)-INSP, Cuernavaca, Mexico

**Keywords:** risk factors, epidemiology, causality, uterine cervical neoplasms, HPV

## Abstract

**Introduction:**

Cervical cancer (CC) is the fourth most prevalent female cancer globally. Understanding its epidemiology is crucial for devising practical strategies suited to geographic and social contexts to attain the global eradication of CC. Hence, this study examined the latest evidence of risk factors contributing to CC development.

**Methods:**

An independent literature search was conducted on PubMed using MESH terms. The primary sources were meta-analyses published from 2010 to 2023, which detail updated evidence on risk factors associated with CC. Additionally, the quality of the evidence was evaluated using the GRADE system and recommendations were made accordingly.

**Results:**

The main risk factors related to the cause of CC include co-infections with other sexually transmitted infections, genetic markers, cervicovaginal microbiota, nutritional factors, comorbidities that affect the immune response, smoking, and the use of hormonal contraceptives with a quality evidence based on the GRADE scale moderate.

**Conclusions:**

Since the necessary cause for CC is persistent cervicovaginal HPV, all the risk factors implicated in the causality of CC act as non-independent cofactors that increase the risk of CC. Thus, changes in public policies aimed at addressing these risk factors are highly recommended and can substantially decrease the risk of CC.

## 1 Introduction

Cervical cancer (CC) is the fourth most prevalent form of cancer among women worldwide. In 2020, the age-standardized incidence rate was 13.3 cases per 100,000 woman-years, and the mortality rate stood at 7.3 deaths per 100,000 woman-years (1). Sub-Saharan Africa, Latin America, and Asia—regions with countries possessing a low human development index—have the highest incidence and mortality rates (2, 3). This discrepancy predominantly stems from the absence of extensive screening programs and insufficient healthcare infrastructure. Conversely, countries with well-established screening initiatives have seen a significant decline in CC cases (4).

In Mexico, 9,439 new CC cases were reported in 2020 (constituting 4.8% of total cases) and 4,335 deaths, with an estimated prevalence of 25,026 cases (5). Approximately 77% of women receive a diagnosis in locally advanced stages, 16% in early stages, and 7% in advanced stages (6). While mortality rates from this cancer have been decreasing since 2001 in the country’s central region, the highest mortality rates are found in some of the most marginalized states, such as Chiapas, Tabasco, and Morelos (7).

A persistent infection with high-risk human papillomavirus (HPV) is the leading cause of CC development. There exist certain factors that increase the potential for exposure to and acquisition of an HPV infection at the cervicovaginal level, as well as structural elements that make a woman more susceptible to CC.

As the female cancer is the greatest prevention potential (8, 9), understanding the etiology of CC is crucial in response to the Global Initiative to eradicate CC as a public health issue (10). Given this context, this study aimed to analyze and present the available evidence of risk factors associated with the development of CC.

## 2 Methods

We conducted a scoping review of meta-analyses published from 2010–2023 in the MEDLINE database via the PubMed database. The search criteria consisted of combined MeSH terms: “risk factors,” “smoking,” “contraceptives,” “genetic markers,” “microbiota,” “immunity,” and “uterine cervical neoplasms.” The Boolean operator “AND” was applied to link the search terms and answer the question: “What is the updated evidence on risk factors associated with cervical cancer?” The search was limited to full-text articles published in English. The quality of the evidence presented in these meta-analyses was assessed as high, moderate, low, or very low according to the Grading of Recommendations Assessment, Development, and Evaluation (GRADE) system. Recommendations, based on the strength of evidence, were made by members of the Mexican National Consensus on Cervical Cancer Epidemiology.

## 3 Results

**Figure 1:**
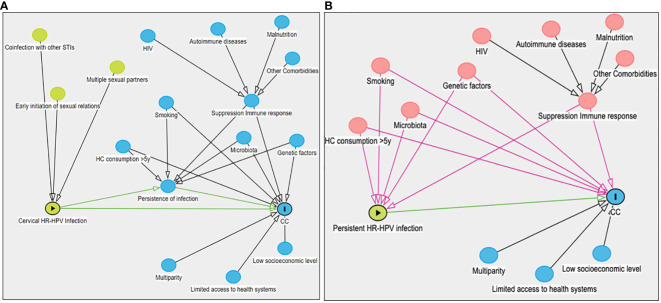
A directed acyclic diagram that represents the causal structure of cervical cancer. This directed acyclic diagram illustrates the exposure variable and the interplay of other risk factors affecting CC causality. The variables are represented by nodes (circles), while the arrows represent the causal direction between variables. Green arrows represent open causal pathways, black arrows symbolize closed non-causal pathways, and pink arrows indicate open non-causal pathways. **(A)** exhibits risk factors associated with the onset of HPV-HR infection (depicted by green circles). Blue circles with bidirectional arrows denote common causes predisposing to HPV-HR infection persistence and CC development. Blue circles with a single arrow represent factors linked to the suppression of the immune response and CC progression. Furthermore, infection persistence is considered a mediator in the association between HPV-HR infection and CC. Sexual history-related risk factors that can increase the likelihood of HPV-HR infection. Lifestyle-related factors such as smoking and use of hormonal contraceptives for periods (>5 years), along with the host’s intrinsic features such as genetic factors, cervicovaginal microbiota, and immune response elements, are linked to a higher likelihood of HPV-HR infection persistence. Structural factors related to social determinants impacting women’s health, particularly in countries like Mexico, are associated with a higher incidence of CC. **(B)** displays the variables associated with exposure and the outcome; however, they are not part of the causal chain (confounders). These confounding variables (revealed as red circles) must be controlled during the study design or adjusted during data analysis to prevent spurious associations when examining the relationship between HPV-HR infection persistence and CC. HR-HPV, high-risk human papillomavirus; CC, cervical cancer; STI, sexually transmitted infection; HIV, human immunodeficiency virus; HC, hormone contraceptives; y, years.

This review provides an update on the evidence found in the literature regarding the association of risk factors with biological plausibility for CC. Given the essential role that persistent high-risk human papillomavirus (HR-HPV) infection at the cervical level plays in the development of CC, we examined the factors associated with HR-HPV infection (exposure variable), the persistence of HR-HPV infection (intermediate phenotype), and the specific outcome of CC (**Figure 1**). The analyses of evidence analysis using the GRADE approach on risk factors related to the causality of CC are listed in **Table 1**.

**Table 1:** Analysis of evidence using the GRADE system by risk factors related to the causality of cervical cancer.

Risk factor	Associated outcome	Reference	Association (IC95%)	*p*	Heterogeneity (I2)	*p*	Bias	*p*	Level of evidence
Sexual life									
STI co-infection									
*Chlamydia*	CC	Bhuvanendran P 2022	OR:2.13 (1.78-2.54)	0.00001	1%	NA	NA	NA	M
*Chlamydia*-HPV co-infection	CC		OR: 2.15 (0.29-15.63)	0.45	89%	NA	NA	NA	L
HSV-2	CC	Li XY 2023	OR: 1.21(1.04-1.41)	0.015	0	>0.05	Egger	0.42	M
*Chlamydia*	CC		OR: 2.21 (1.62-3.03)	0.0000	45.6%	NA	Egger	0.0418	M
*Chlamydia*	CC		OR:2.19 (1.74-2.74)	1.28 x 10-11	47.4%	<10^-6^	Egger	0.0317	M
*Chlamydia*-HPV co-infection	CC		OR: 4.37 (2.75–6.96)	4.593 x 10-10	44%	<10^-6^	Egger	0.0054	L
*Chlamydia*	HPV infection	Naldini G 2019	OR:2.12 (1.80-2.49)	<0.0001	82.7	<0.0001	Egger	0.0001	L
Endogenous factors									
Genetic factors									
CTLA4 A/G (rs231775) Allele model	CC	Zhang X 2014	OR: 1.13 (1.03-1.25)	0.01	0%	0.44	NA		M
IFN-gamma rs2430561 Heterozygous model			OR: 0.76 (0.60-0.95)	0.03	0%	0.38	NA		M
HLA-DQA1 0201 Carriers vs. no carriers			OR: 0.59 (0.47-0.73)	0.019	0%	0.6	NA		M
HLA-DQB1 0603 Carriers vs. no carriers			OR: 0.70 (0.56-0.89)	0.0001	0%	0.53	NA		M
BRIP1 rs2048718 Dominant model		Martínez-Nava GA 2016	OR: 0.80 (0.67-0.95)	0.01	0%	0.99	NA		M
BRIP1 rs11079454 Recessive model			OR: 0.79 (0.63–0.99)	0.04	0%	0.99	NA		M
p21 rs1801270 Heterozygous model			OR: 0.80 (0.66–0.98)	0.03	0%	0.37	NA		M
p53 rs1042522 Dominant model			OR: 1.28 (0.98–1.66)	0.07	35%	0.04	Egger	<0.1	L
IL-1B −511 C > T (rs16944) Co-dominant model		de Moura EL 2021	OR: 1.46 (1.03–2.08)	0.03	0%	0.64	NA		M
IL-6 − 174 G > C (rs1800795) Recessive model			OR:1.42 (1.03–1.95)	0.03	0%	0.59	NA		M
TNFA −238 G > A (rs361525) Recessive model			OR: 4.10 (1.16–14.48)	0.03	0%	0.77	NA		M
TNFA −308 G > A (rs1800629) Dominant model			OR: 1.20 (1.04–1.38)	0.01	31%	0.2	Egger	<.0001	L
IL-17A −197 G > A (rs2275913) Dominant model			OR: 1.55 (1.33–1.82)	<0.00001	0%	0.97	Egger	<.0001	L
Cervical microbiota									
*Lactobacillus iners* vs. *L. crispatus*	Persistent HPV infection	Brusselaers N 2019	RR: 1.06 (0.42-2.63)	<0.05	0%	0.46	NA		M
*Lactobacillus iners* vs. *L. crispatus*			RR: 2.00 (1.05-3.81)	<0.05	0%	0.39	NA		M
Predominant in LSIL vs. normal cervix	Dysplasia - CC		RR: 2.01 (1.40-3.01)	<0.05	0%	0.76	NA		M
Vaginal dysbiosis	Persistent HPV infection		RR: 1.33 (1.18-1.50)	<0.05	0%	0.62	NA		M
Vaginal dysbiosis			RR: 1.14 (1.01-1.28)	<0.05	44%	0.096	NA		L
Cervicovaginal microbiota dominated by *Lactobacillus* spp. among cases with HR-HPV vs. controls		Wang H 2019	OR: 0.64 (0.48-0.87)	<0.05	6%	0.39	NA		M
Cervicovaginal microbiota dominated by *Lactobacillus* spp. among cases with HR-HPV vs. controls	CIN		OR: 0.53 (0.34-0.83)	<0.05	0%	0.57	NA		M
Cervicovaginal microbiota dominated by *Lactobacillus* spp. between cases with CIN vs. controls	CC		OR: 0.12 (0.04-0.36)	<0.05	0%	0.59	NA		M
Cervicovaginal microbiota dominated by *Lactobacillus* spp. between cases with CIN vs. controls	HPV infection		OR: 0.96 (0.69-1.34)	<0.05	0%	0.53	NA		M
Cervicovaginal microbiota dominated by *Lactobacillus* spp. between cases with CIN vs. controls	CIN		OR: 0.99 (0.60-1.64)	<0.05	0%	0.5	NA		M
Cervicovaginal microbiota dominated by *Lactobacillus* spp. between cases with CIN vs. controls	CC		OR: 0.13 (0.02-1.13)	<0.05	0%	1.0	NA		M
Cervicovaginal microbiota dominated by *Lactobacillus* spp. between cases with CIN vs. controls	HPV infection		OR: 0.49 (0.31-0.79)	<0.05	10%	0.35	NA		M
Cervicovaginal microbiota dominated by *Lactobacillus* spp. between cases with CIN vs. controls	CIN		OR: 0.50 (0.29-0.88)	<0.05	0%	0.87	NA		M
Cervicovaginal microbiota dominated by *Lactobacillus* spp. between cases with CIN vs. controls	CC		OR: 0.17 (0.03-1.05)	<0.05	0%	0.65	NA		M
Comorbidities									
HIV	CC	Liu G 2018	RR: 4.1 (2.3-6.6)	–	–	–	–	–	L
Gestational diabetes	CC	Wang Y 2020	RR: 1.02 (0.81-1.29)	0.843	0%	0.552	–	–	L
Autoimmunities									
SLE	CC	Chen Y 2021	RR: 6.01 (1.45-24.87)	–	76.90%	0.013	Begg, Egger	0.805, 0.615	L
SLE	CC	Clarke AE 2021	RR 1.66 (1.16-2.36)	–	77%	<0.001	Egger	≥0.05	L
Rheumatoid arthritis	CC	Simon TA 2015	RR: 0.87 (0.72-1.05)	–	–	–	–	–	L
Nutritional Factors									
Low vs. high blood vitamin A	CC	Zhang X 2012	OR: 1.14 (0.83-1.56)	0.422	0%	0.96	Egger	0.249	L
Low vs. high blood vitamin E	CC	Hu X 2017	OR: 0.52 (0.4-0.69)	<0.001	86%	<0.0001	Begg, Egger	0.53, 0.322	L
Low vs. high blood carotene	CC	Zhang X 2012	OR: 0.48 (0.3-0.77)	0.002	69%	0.01	–	–	L
Low vs. high blood selenium	CC	He D 2017	OR: 0.55 (0.42-0.73)	<0.001	0%	0.657	Egger	0.691	L
Overweight	CC	Poorolajal J 2016	OR 1.03 (0.81-1.25)	0.146	21.2%	0.268	Begg, Egger	0.835, 0.945	L
Obesity	CC	Poorolajal J 2016	OR: 1.1 (1.03-1.17)	0.001	13.7%	0.326	Begg, Egger	0.404, 0.169	M
Exogenous factors									
Smoking									
Frequent smoker vs. never smoked	CC	Malevolti MC 2023	RR: 1.67 (1.47-1.89)	>0.05	75%	< 0.01	NA		L
			RR: 1.70 (1.53-1.88)	>0.05	70%	< 0.02	NA		L
Current smoker vs. never smoked			RR: 1.15 (1.02; 1.29)	>0.05	43%	0.05	NA		L
			RR: 1.13 (1.02; 1.24)	>0.05	26%	0.13	NA		L
Passive smoking subgroup analysis (LSIL)	LSIL	Zeng XT 2012	OR: 1.43 (1.11-1.84)	0.01	0%	0.87	Egger test	p<0.001	M
Passive smoking subgroup analysis (CC)	CC		OR: 2.77 (1.85-4.17)	<0.001	53%	0.08	Egger test	p<0.001	L
Hormone contraceptives									
Oral contraceptive consumption	Invasive CC	Asthana S 2020	OR: 1.59 (1.31-1.93)	<0.00001	62%	0.002	NA	NA	M
Oral contraceptive consumption	CC	Peng Y 2017 May	OR: 1.12 (0.90-1.38)	>0.05	82.8%	0.01	Begg	0.49	L

STI, Sexual transmitted infection; CTL, cytotoxic T lymphocytes; IFN, interferon; HLA, human leukocyte antigens; IL, interleukin; TNF, tumor necrosis factor; LSIL, low-grade squamous intraepithelial lesion; CIN, cervical intraepithelial neoplasia; SLE, systemic lupus erythematosus; CC, cervical cancer; RR, relative risk; OR, odds ratio; CI, confidence interval; I2, I-square statistic in meta-analysis; L, low; M, moderate. NA, does not apply.

### 3.1 Risk factors associated with a greater probability of HR-HPV infection at the cervical level

#### 3.1.1 Number of sexual partners

Studies have observed that an increased number of sexual partners correlates with a heightened risk of obtaining an abnormal Pap smear result [odds ratio (OR): 5.5] (11). This risk for HPV infection similarly escalates with increasing numbers of sexual partners (12). In Peru, reports indicated a higher risk of HPV infection among individuals who had more than five sexual partners throughout their lifetime (13). Similarly, researchers in the United States and China found an association between HPV infection and having two or more sexual partners (14). A rise in risk with multiple partners (OR: 1.91) and high-risk genotypes was also reported in Tunisia (15). Moreover, studies in Mexico revealed that having more than five sexual partners heightened the risk for HPV-16 and non-HPV 16/18 infection (16).

#### 3.1.2 Age of onset of sexual life

Another extensively researched risk factor concerning sexual history is engaging in sexual activity at an early age. A study conducted in China found that the risk of HPV infection increased when sexual activity commenced at 19 years old or younger (OR: 1.51) (17). Similarly, researchers in Peru noted an elevated risk of HPV infection (OR: 1.4) when sexual relations began at an age younger than 18 years (13).

#### 3.1.3 Sexually transmitted co-infections

##### 3.1.3.1 Coinfection with *Chlamydia*

A meta-analysis found an increased risk for CC associated with *Chlamydia* infection (OR: 1.96) (18), a finding consistent with another meta-analysis (OR: 2.21) (19). The same meta-analysis suggested an even greater increase in CC risk with concurrent *Chlamydia* and HPV infections (OR: 2.13) (18). Separate studies have also reported *Chlamydia* to be more prevalent in HPV-positive women compared to those who are HPV-negative (20). Furthermore, in women with *Chlamydia*, the risk for HPV infection increases (OR: 2.21) (21). In addition, the literature has shown that past *Chlamydia* infection is a risk factor for contracting HPV (OR: 1.72) (21).

##### 3.1.3.2 Co-infection with herpes simplex virus

A study in Mexico on herpes simplex virus (HSV-2) documented that the likelihood of having an active HSV-2 infection in HR-HPV-positive cases was nine times higher than in negative cases (*p* = 0.03). Furthermore, the primary factors related to an active HSV-2 infection were a history of risky sexual behavior and HR-HPV infection (22).

### 3.2 Risk factors associated with a higher likelihood of persistent HPV infection at the cervical level

#### 3.2.1 Endogenous factors

##### 3.2.1.1 Genetic factors

Most studies examining genetic factors as risk factors for CC have been association studies focusing on single nucleotide polymorphisms (SNPs) within candidate genes involved in oncogenesis and cellular immune response (23). These studies relate primarily to immune response evasion in patients persistently infected with HPV and CC (24, 25).

To date, few comprehensive meta-analyses have investigated the association between genetic polymorphisms unrelated to a specific biological pathway and the risk of CC. These studies primarily focus on the evidence reported in the existing literature (26–29). A recent meta-analysis of studies that examined the association of SNPs in genes coding for cytokines found SNPs in IL-17A, IL-17F, IL-12A, IL-12B, TNFA, IL-1B, IL-6, and IL-10 (26). Another meta-analysis focusing exclusively on case-control studies reported a negative association between CC and a polymorphism of the p21 gene, a potent cell proliferation and DNA replication inhibitor, as well as two polymorphisms of the BRIP1 gene, a crucial gene in the BRCA-associated DNA repair process (27). In contrast, the meta-analyses of Wang et al. (29) and Zhang et al. (28) reported divergent SNPs significantly associated with the risk of developing CC, potentially due to differences in defined inclusion criteria.

##### 3.2.1.2 Cervicovaginal microbiota

Emerging evidence suggests that increased diversity of the vaginal microbiota, coupled with a reduced relative abundance of *Lactobacillus* spp. may play a role in the acquisition and persistence of HPV, as well as the development of precancer and CC (30, 31). There are only a few published meta-analyses to date that focus on the results from studies exploring the causal relationship between vaginal microbiota and CC (32, 33). The 2019 meta-analysis by Brusselaers et al. reported an association between vaginal dysbiosis and a higher risk of HPV incidence (relative risk [RR]: 1.33), HPV persistence (RR: 1.14), high-grade lesions, and CC (RR: 2.01) (33). In contrast, the 2019 meta-analysis by Wang et al. focused solely on the relationship between cervicovaginal microbiota dominated by *Lactobacillus* spp. and HR-HPV, cervical intraepithelial neoplasia (CIN), and CC infection using data from cross-sectional studies (32). This analysis reported a protective association related to the detection of HR-HPV infection (OR: 0.64), CIN (OR: 0.53), and CC (OR: 0.12) (32).

##### 3.2.1.3 Nutritional factors

###### 3.2.1.3.1 Vitamins and minerals

Given that malnutrition exists in some patients at the time of CC diagnosis, it is conceivable that nutritional deficiencies may contribute to the disease’s pathogenesis, given their intimate association with the immune system. Some of the nutrients potentially involved include vitamins A, C, D, and E, calcium, and various antioxidants (34). Nevertheless, one meta-analysis found elevated beta-carotene levels in the blood to be protective against CC development (OR: 0.48) in stark contrast to high vitamin A levels (35). In another study, consumption of over 502.6 mg/dL of calcium (OR: 0.54) and more than 291 IU of vitamin D (OR: 0.51) was identified as a protective factor against the development of invasive CC, except in individuals who smoke or consume alcohol (36). Other meta-analyses reported higher blood levels of vitamin E (OR: 0.52) and selenium (OR: 0.55) associated with protective effects against CC (37, 38).

###### 3.2.1.3.2 Other nutritional indicators

Patients recently diagnosed with CC may exhibit lower circulating levels of non-enzymatic antioxidants (e.g., glutathione) and enzymatic antioxidants (e.g., glutathione S transferase, glutathione peroxidase, and superoxide dismutase). They may also have lower levels of vitamins C and E compared to patients who do not have cancer. These discrepancies could be attributed to the more significant elimination of lipid peroxides and the sequestration of glutathione by tumor cells (39). In addition, another meta-analysis revealed that while being overweight did not have a significant association with CC, obesity did have a slight correlation (OR: 1.1) (40).

##### 3.2.1.4 Comorbidities that condition the immune response

###### 3.2.1.4.1 Acquired immunodeficiency virus

Acquired immunodeficiency virus (HIV) infection significantly accelerates carcinogenesis in the progression of HPV infection. Studies have found that women with HIV are at a higher risk of contracting and spreading HPV (41, 42), predominantly when their CD4 count decreases (42). This demographic has a heightened incidence of both low- and high-grade squamous intraepithelial lesions and an elevated risk for developing CC, commonly as a result of HPV strains 16 and 18 (42–44). A recent meta-analysis found that women with HIV have a 4.1 higher risk of developing CC than women without HIV (RR: 4.1) (42).

###### 3.2.1.4.2 Systemic lupus erythematosus

Systemic lupus erythematosus (SLE) can lead to a dysregulated immune system, potentially prompting persistent HPV infection and subsequent CC development. Women with SLE have an increased risk of HPV infection (45), developing cervical atypia (46), accruing low-grade squamous intraepithelial lesions, and CC (46, 47). The connection between SLE and CC development was substantiated in two independent meta-analyses, both of which indicated an increased risk for CC (46, 47).

###### 3.2.1.4.3 Other comorbidities

Gestational diabetes and rheumatoid arthritis have not been found to increase the risk of CC (48, 49). Furthermore, HPV infection has not been shown to be associated with immunosuppressive therapy or any other treatment (49).

#### 3.2.2 Exogenous factors

##### 3.2.2.1 Smoking

Epidemiological studies have suggested a dose-response relationship between cervical neoplasia/CC and smoking, a proposition partially corroborated by experimental studies (50). Malevolti and collaborators performed a meta-analysis that reported a combined RR of preinvasive lesions and CC for current smokers (RR: 2.11) versus never-smokers (RR: 1.70) and for former smokers (RR: 1.29) versus never-smokers (RR: 1.13). The risk increases to over 2 with a habit of approximately 20 cigarettes/day or 15 pack-years for invasive CC and about nine cigarettes/day or eight pack-years for preinvasive lesions. However, the risk subsides about 15 years after cessation of smoking (51). In terms of passive smoking as a risk factor for CC, the results are inconsistent. A multicenter case-control study did not identify passive smoking as a risk factor for invasive CC (52). Nonetheless, a separate case-control meta-analysis reported a 73% heightened risk of CC (53).

##### 3.2.2.2 Extended use of hormonal contraceptives

In a study by Gadducci et al. (54), an increased incidence of CC was reported in users of oral contraceptives for periods of 5–9 years (RR: 1.3–1.6) and 10 years (RR 2.2–2.5). These findings corroborate a meta-analysis that reported an increased OR with oral contraceptive use for 2–5 years (OR: 1.36), >5 years (OR: 1.93), and >10 years (OR: 2.24) (55). Another study reported a significant association between oral contraceptive use for 15 years and higher risk of CIN3/cancer *in situ* (hazard ratio [HR]: 1.6) and invasive CC (HR: 1.8) compared to non-use. Former menopausal hormone therapy use was associated with a reduced risk of invasive CC (HR: 0.5). A restricted analysis of HPV-seropositive cases and controls revealed an inverse association between intrauterine device use and CIN3 (56). A meta-analysis examining the relationship between hormonal contraceptives and the risk of CC in various ethnic groups found no link between Caucasian, African, and mixed populations’ oral contraceptive use and CC, although there was a higher risk for CC in Asian women (OR: 1.43) (57).

### 3.3 Structural risk factors related to CC

Socioeconomic factors, while not direct causes of CC, can increase a woman’s susceptibility to it. Women with low socioeconomic status (SES), residing in rural areas, or with limited education often delay medical care, thereby elevating their risk levels (58–61). Evidence has also shown that a lower SES correlates with a higher chance of advanced CC (62, 63). One Turkish study discovered a linear relationship between education level, understanding of CC, and coping capacity for certain situations (64). In Ethiopia, lower levels of education also corresponded to limited knowledge of CC (65). Meanwhile, in Uganda, less education was associated with a higher risk of contracting high-risk human papillomavirus (HR-HPV) (66). In Canada, there were more CC cases in rural and poorer areas than in urban or affluent locations (67). A meta-analysis suggested that low SES (OR: 2.68) or a low level of education (OR: 1.97) increases the risk of developing CC (68).

One strength of this study is its synthetic and updated review of risk factors related to the causality of CC, which will be valuable to individuals interested in epidemiology and causality analysis. Nevertheless, the main limitation is that some meta-analyses about the causes of CC are based on primary observational studies with a high risk of bias.

## 4 Level of evidence conclusions

- The requisite cause for CC is both the presence and carcinogenic activity of HPV. Consequently, all supplementary risk factors reviewed function as non-independent co-factors in CC causality.
- Notably, the most investigated risk factors, presenting moderate evidence of potentially independent risk factors for CC, include smoking (i.e., exogenous factors) and the prolonged use of hormonal contraceptives.
- There is a considerable risk of bias in associating smoking and the risk of cervical neoplasia and CC development. This is due to reliance on observational studies featuring inadequate adjustment of prognostic factors, vague descriptions of the study population and clinical outcomes, a blend of intraepithelial neoplasia and CC as outcome measures, and the utilization of different control groups.
- Moreover, few studies have examined the link between CC and the use of hormonal contraceptives, calling for improved control of confounding variables. The most consistent evidence relates to hormonal contraceptive usage duration, particularly periods of 5 or more years.
- Given the cervicovaginal microbiota and comorbidities impacting the immune response, the causality evidence for CC is generally low.
- Despite the consistent connections shown by polymorphisms studied in CC, their small magnitude requires more replication studies on CC susceptibility variants.
- The cervicovaginal microbiota has been proposed as a crucial local immune response modifier, promoting the removal or persistence of HR-HPV infection and the risk of progression to malignancy. There is moderate evidence associating HPV infection and genital dysbiosis, although positive associations may reflect residual confounding due to unmeasured sexual risk behaviors.
- Risk factors involving a compromised immune response predispose individuals to a more aggressive course of neoplasia but are not independent CC risk factors.
- Comorbidities influencing a poor immune response and favoring HPV infection persistence include HIV and SLE.
- Nutritional factors influence CC progression response. The predominantly low-quality evidence indicates that dietary components, such as vitamins, minerals, and antioxidants, might aid in eliminating HPV infection, CIN, and even carcinogenesis.
- Sexual lifestyle and structural factors—both contributing to a higher probability of acquiring HPV infection and increasing the likelihood of CC—are risk factors with a low causality evidence level.
- Factors linked to health-disease structural determinants are particular to certain populations. Lower education or socioeconomic status escalates the CC development risk, but causality evidence remains low.
- Sexual history risk factors, such as beginning sexual activity at an early age and having a high number of sexual partners, are associated with a higher likelihood of being exposed to and acquiring HPV infection.
- Co-infection of HPV with other sexually transmitted infections (e.g., *Chlamydia* and HSV-2) is the only known sexual life history risk factor that has been moderately proven to be causally related to cervical cancer.
- An updated evaluation of the available scientific literature evidence via the GRADE system on CC causality and risk factors provides valuable, synthesized information for those interested in CC causality analysis and epidemiology.

## 5 Recommendations

- From a public health standpoint, all the lifestyle-related risk factors examined, irrespective of the proposed causal mechanism being direct or confounding, are modifiable elements that, when reduced, can significantly decrease the risk of cervical cancer. The quality of the supporting evidence is high (GRADE), and the strength of the recommendation is strong.
- The medical literature consistently underscores a scientifically-backed public health message regarding the importance of preventing and eliminating lifestyle factors tied to the causation of cervical cancer. By addressing these factors, the risk of cervical cancer can be reduced. The quality of evidence supporting this is high (GRADE), and the strength of the recommendation is strong.
- Cervical cancer is the type of cancer in women with the highest potential for prevention. That said, there is an urgent need to enhance health promotion efforts, including providing health-related information on the risks of tobacco use and hormonal contraceptive utilization, implementing age- and culture-appropriate sex education, and promoting condom use. The quality of evidence supporting these measures is high (GRADE), and the strength of the recommendation is strong.
- Fortifying health promotion programs offered in primary care units, such as adolescent and youth-friendly services at the national level, is paramount. The quality of supporting evidence is high (GRADE), and the strength of recommendation is strong.
- Increased coverage of the primary prevention program at the national level is necessary. The quality of the supporting evidence is high (GRADE), and the strength of the recommendation is strong.
- It is necessary to ensure adherence to available regulations and official guidelines for HPV infection prevention and CC control, considering HPV infection is the most common sexually transmitted infection leading to CC. The quality of evidence supporting this is high (GRADE), and the strength of recommendation is strong.

## Author contributions

JL: Conceptualization, Data curation, Methodology, Resources, Validation, Writing – original draft, Writing – review & editing. SG: Investigation, Writing – original draft, Writing – review & editing. VG: Investigation, Writing – original draft, Writing – review & editing. KT: Conceptualization, Data curation, Formal analysis, Funding acquisition, Investigation, Methodology, Project administration, Resources, Supervision, Validation, Writing – original draft, Writing – review & editing.

## References

[1] International Agency for Research on Cancer. Cancer today. Cancer incidence and mortality statistics worldwide. Cancer incidence and mortality statistics worldwide. Lyon, France: World Health Organization (2020). Available at: https://gco.iarc.fr/today/data/factsheets/cancers/23-Cervix-uteri-fact-sheet.pdf.

[2] Momenimovahed Z Mazidimoradi A Maroofi P Allahqoli L Salehiniya H Alkatout I. Global, regional and national burden, incidence, and mortality of cervical cancer. Cancer Rep. (2023) 6:e1756. doi: 10.1002/cnr2.1756 PMC10026270 36545760

[3] Bouvard V Wentzensen N Mackie A Berkhof J Brotherton J Giorgi-Rossi P. The IARC perspective on cervical cancer screening. N Engl J Med. (2021) 385:1908–18. doi: 10.1056/NEJMsr2030640 PMC12125667 34758259

[4] Singh D Vignat J Lorenzoni V Eslahi M Ginsburg O Lauby-Secretan B. Global estimates of incidence and mortality of cervical cancer in 2020. Lancet Glob Heal. (2023) 11:e197–206. doi: 10.1016/S2214-109X(22)00501-0 PMC9848409 36528031

[5] International Agency for Research on Cancer. Cancer incidence and mortality statistics in Mexico (2020). Available at: https://gco.iarc.fr/today/data/factsheets/populations/484-Mexico-fact-sheets.pdf.

[6] Torreglosa-Hernández S Grisales-Romero H Morales-Carmona E Hernández-Ávila JE Huerta-Gutiérrez R Barquet-Muñoz SA. Survival analysis and associated factors in patients with cervical cancer financed by the Seguro Popular in Mexico. Salud Publica Mex. (2022) 64:76–86. doi: 10.21149/13119 35438904

[7] Palacio-Mejía LS Hernández-Ávila JE. Tendencia de la tasa de mortalidad según tipo de cáncer, México 2000 - 2020. Unidad de Inteligencia en Salud Pública. Available at: https://uisp.insp.mx/wp/index.php/analisis-de-la-mortalidad-por-cancer/.

[8] Bogdanova A Andrawos C Constantinou C. Cervical cancer, geographical inequalities, prevention and barriers in resource depleted countries (Review). Oncol Lett. (2022) 23:113. doi: 10.3892/ol 35251344 PMC8850967

[9] World Health Organization. WHO guidelines for screening and treatment of cervical pre- cancer lesions for cervical cancer prevention. 2nd. Geneva: World Health Organization (2021). 34314129

[10] World Health Organization. Global strategy to accelerate the elimination of cervical cancer as a public health problem. Geneva: World Health Organization (2020).

[11] Huang Y Wu X Lin Y Li W Liu J Song B. Multiple sexual partners and vaginal microecological disorder are associated with HPV infection and cervical carcinoma development. Oncol Lett. (2020) 20:1915–21. doi: 10.3892/ol PMC7377087 32724435

[12] Vaccarella S Franceschi S Herrero R Muñoz N Snijders PJF Clifford GM. Sexual behavior, condom use, and human papillomavirus: Pooled analysis of the IARC human papillomavirus prevalence surveys. Cancer Epidemiol Biomarkers Prev. (2006) 15:326–33. doi: 10.1158/1055-9965.EPI-05-0577 16492924

[13] Almonte M Ferreccio C Gonzales M Delgado JM Buckley CH Luciani S. Risk factors for high-risk human papillomavirus infection and cofactors for high-grade cervical disease in Peru. Int J Gynecol Cancer. (2011) 21:1654–63. doi: 10.1097/IGC.0b013e3182288104 21892094

[14] Yang H Xie Y Guan R Zhao Y Lv W Liu Y. Factors afecting HPV infection in U.S. and Beijing females: A modeling study. Front Public Heal. (2022) 10:1052210. doi: 10.3389/fpubh.2022.1052210 PMC9794849 36589946

[15] Ardhaoui M Letaief H Ennaifer E Bougatef S Lassili T Bel Haj Rhouma R. The prevalence, genotype distribution and risk factors of human papillomavirus in Tunisia: A national-based study. Viruses. (2022) 14:2175. doi: 10.3390/v14102175 36298732 PMC9611589

[16] Torres-Poveda K Ruiz-Fraga I Madrid-Marina V Chavez M Richardson V. High risk HPV infection prevalence and associated cofactors: A population-based study in female ISSSTE beneficiaries attending the HPV screening and early detection of cervical cancer program. BMC Cancer. (2019) 19:1205. doi: 10.1186/s12885-019-6388-4 31823749 PMC6905062

[17] Yang D Zhang J Cui X Ma J Wang C Piao H. Risk factors associated with human papillomavirus infection, cervical cancer, and precancerous lesions in large-scale population screening. Front Microbiol. (2022) 13:914516. doi: 10.3389/fmicb.2022.914516 35847094 PMC9282163

[18] Bhuvanendran Pillai A Mun Wong C Dalila Inche Zainal Abidin N Fazlinda Syed Nor S Fathulzhafran Mohamed Hanan M Rasidah Abd Ghani S. *Chlamydia* infection as a risk factor for cervical cancer: A systematic review and meta-analysis. Iran J Public Heal. (2022) 51:508–17. doi: 10.18502/ijph.v51i3.8926 PMC9276600 35865072

[19] Zhu H Shen Z Luo H Zhang W Zhu X. *Chlamydia* trachomatis infection-associated risk of cervical cancer: A meta-analysis. Med (United States). (2016) 95:e3077. doi: 10.1097/MD.0000000000003077 PMC4998531 27043670

[20] Karim S Souho T Benlemlih M Bennani B. Cervical cancer induction enhancement potential of *Chlamydia* trachomatis: A systematic review. Curr Microbiol. (2018) 75:1667–74. doi: 10.1007/s00284-018-1439-7 29356877

[21] Naldini G Grisci C Chiavarini M Fabiani R. Association between human papillomavirus and *Chlamydia* trachomatis infection risk in women: a systematic review and meta-analysis. Int J Public Health. (2019) 64:943–55. doi: 10.1007/s00038-019-01261-w 31175391

[22] Bahena-Román M Sánchez-Alemán MA Contreras-Ochoa CO Lagunas-Martínez A Olamendi-Portugal M López-Estrada G. Prevalence of active infection by herpes simplex virus type 2 in patients with high-risk human papillomavirus infection: A cross-sectional study. J Med Virol. (2020) 92:1246–52. doi: 10.1002/jmv.25668 31925791

[23] Torres-Poveda K Burguete-García AI Bahena-Román M Méndez-Martínez R Zurita-Díaz MA López-Estrada G. Risk allelic load in Th2 and Th3 cytokines genes as biomarker of susceptibility to HPV-16 positive cervical cancer: A case control study. BMC Cancer. (2016) 16:330. doi: 10.1186/s12885-016-2364-4 27220278 PMC4879749

[24] Wang K Jiao Z Chen H Liu X Lu J Liu X. The association between rs1800872 polymorphism in interleukin-10 and risk of cervical cancer: A meta-analysis. Med (United States). (2021) 100:E23892. doi: 10.1097/MD.0000000000023892 PMC7837934 33545957

[25] Vanajothi R Srikanth N Vijayakumar R Palanisamy M Bhavaniramya S Premkumar K. HPV-mediated cervical cancer: A systematic review on immunological basis, molecular biology, and immune evasion mechanisms. Curr Drug Targets. (2022) 23:782–801. doi: 10.2174/1389450123666211221160632 34939539

[26] de Moura EL dos Santos ACM da Silva DM dos Santos BB Figueredo D de S Moura AWA. Association of Polymorphisms in Cytokine genes with susceptibility to Precancerous Lesions and Cervical Cancer: A systematic review with meta-analysis. Immunol Invest. (2021) 50:492–526. doi: 10.1080/08820139.2020.1778023 32602796

[27] Martínez-Nava GA Fernández-Niño JA Madrid-Marina V Torres-Poveda K. Cervical cancer genetic susceptibility: A systematic review and meta-analyses of recent evidence. PloS One. (2016) 11:e0157344. doi: 10.1371/journal.pone.0157344 27415837 PMC4945039

[28] Zhang X Zhang L Tian C Yang L Wang Z. Genetic variants and risk of cervical cancer: Epidemiological evidence, meta-analysis and research review. BJOG Int J Obstet Gynaecol. (2014) 121:664–73. doi: 10.1111/1471-0528.12638 24548744

[29] Wang S Sun H Jia Y Tang F Zhou H Li X. Association of 42 SNPs with genetic risk for cervical cancer: An extensive meta-analysis. BMC Med Genet. (2015) 16:25. doi: 10.1186/s12881-015-0168-z 25928231 PMC4436168

[30] Mitra A MacIntyre DA Marchesi JR Lee YS Bennett PR Kyrgiou M. The vaginal microbiota, human papillomavirus infection and cervical intraepithelial neoplasia: What do we know and where are we going next? Microbiome. (2016) 4:58. doi: 10.1186/s40168-016-0203-0 27802830 PMC5088670

[31] Audirac-Chalifour A Torres-Poveda K Bahena-Román M Téllez-Sosa J Martínez-Barnetche J Cortina-Ceballos B. Cervical microbiome and cytokine profile at various stages of cervical cancer: A pilot study. PloS One. (2016) 11:e0153274. doi: 10.1371/journal.pone.0153274 27115350 PMC4846060

[32] Wang H Ma Y Li R Chen X Wan L Zhao W. Associations of cervicovaginal lactobacilli with high-risk HPV infection, cervical intraepithelial neoplasia, and cancer: a systematic review and meta-analysis. J Infect Dis. (2019) 220:1248–58. doi: 10.1093/infdis/jiz325 31242505

[33] Brusselaers N Shrestha S van de Wijgert J Verstraelen H. Vaginal dysbiosis and the risk of human papillomavirus and cervical cancer: systematic review and meta-analysis. Am J Obstet Gynecol. (2019) 221:9–18.e8. doi: 10.1016/j.ajog.2018.12.011 30550767

[34] Tomita LY Longatto Filho A Costa MC Avilla Andreoli MA Villa LL Franco EL. Diet and serum micronutrients in relation to cervical neoplasia and cancer among low-income Brazilian women. Int J Cancer. (2010) 126:703–14. doi: 10.1002/ijc.24793 19642096

[35] Zhang X Dai B Zhang B Wang Z. Vitamin A and risk of cervical cancer: A meta-analysis. Gynecol Oncol. (2012) 124:366–73. doi: 10.1016/j.ygyno.2011.10.012 22005522

[36] Hosono S Matsuo K Kajiyama H Hirose K Suzuki T Kawase T. Association between dietary calcium and vitamin D intake and cervical carcinogenesis among Japanese women. Eur J Clin Nutr. (2010) 64:400–9. doi: 10.1038/ejcn.2010.28 20197786

[37] Hu X Li S Zhou L Zhao M Zhu X. Effect of vitamin E supplementation on uterine cervical neoplasm: A meta-analysis of case-control studies. PloS One. (2017) 12:e0183395. doi: 10.1371/journal.pone.0183395 28829815 PMC5567498

[38] He D Wang Z Huang C Fang X Chen D. Serum selenium levels and cervical cancer: systematic review and meta-analysis. Biol Trace Elem Res. (2017) 179:195–202. doi: 10.1007/s12011-017-0982-6 28255860

[39] Manju V Sailaja JK Nalini N. Circulating lipid peroxidation and antioxidant status in cervical cancer patients: A case-control study. Clin Biochem. (2002) 35:621–5. doi: 10.1016/S0009-9120(02)00376-4 12498996

[40] Poorolajal J Jenabi E. The association between BMI and cervical cancer risk: A meta-analysis. Eur J Cancer Prev. (2016) 25:232–8. doi: 10.1097/CEJ.0000000000000164 25932869

[41] Clifford GM De Vuyst H Tenet V Plummer M Tully S Franceschi S. Effect of HIV infection on human papillomavirus types causing invasive cervical cancer in africa. J Acquir Immune Defic Syndr. (2016) 73:332–9. doi: 10.1097/QAI.0000000000001113 PMC5172520 27331659

[42] Liu G Sharma M Tan N Barnabas RV. HIV-positive women have higher risk of human papilloma virus infection, precancerous lesions, and cervical cancer. Aids. (2018) 32:795–808. doi: 10.1097/QAD.0000000000001765 29369827 PMC5854529

[43] Abraham AG D’Souza G Jing Y Gange SJ Sterling TR Silverberg MJ. Invasive cervical cancer risk among HIV-infected women: A North American multicohort collaboration prospective study. J Acquir Immune Defic Syndr. (2013) 62:405–13. doi: 10.1097/QAI.0b013e31828177d7 PMC3633634 23254153

[44] Clifford GM Tully S Franceschi S. Carcinogenicity of human papillomavirus (HPV) types in HIV-positive women: A meta-analysis from HPV infection to cervical cancer. Clin Infect Dis. (2017) 64:1228–35. doi: 10.1093/cid/cix135 PMC5399941 28199532

[45] González LA Alarcón GS. The evolving concept of SLE comorbidities. Expert Rev Clin Immunol. (2017) 13:753–68. doi: 10.1080/1744666X.2017.1327353 28471690

[46] Chen Y Wu X Liu L. Association between systemic lupus erythematosus and risk of cervical atypia: A meta-analysis. Lupus. (2021) 30:2075–88. doi: 10.1177/09612033211048129 34715754

[47] Clarke AE Pooley N Marjenberg Z Langham J Nicholson L Langham S. Risk of Malignancy in patients with systemic lupus erythematosus: Systematic review and meta-analysis. Semin Arthritis Rheumatol. (2021) 51:1230–41. doi: 10.1016/j.semarthrit.2021.09.009 34710720

[48] Wang Y Yan P Fu T Yuan J Yang G Liu Y. The association between gestational diabetes mellitus and cancer in women: A systematic review and meta-analysis of observational studies. Diabetes Metab. (2020) 46:461–71. doi: 10.1016/j.diabet.2020.02.003 32097717

[49] Simon TA Thompson A Gandhi KK Hochberg MC Suissa S. Incidence of Malignancy in adult patients with rheumatoid arthritis: A meta-analysis. Arthritis Res Ther. (2015) 17:212. doi: 10.1186/s13075-015-0728-9 26271620 PMC4536786

[50] Wei L Griego AM Chu M Ozbun MA. Tobacco exposure results in increased E6 and E7 oncogene expression, DNA damage and mutation rates in cells maintaining episomal human papillomavirus 16 genomes. Carcinogenesis. (2014) 35:2373–81. doi: 10.1093/carcin/bgu156 PMC4178472 25064354

[51] Malevolti MC Lugo A Scala M Gallus S Gorini G Lachi A. Dose-risk relationships between cigarette smoking and cervical cancer: a systematic review and meta-analysis. Eur J Cancer Prev. (2023) 32:171–83. doi: 10.1097/CEJ.0000000000000773 36440802

[52] Louie KS Castellsague X De Sanjos S Herrero R Meijer CJ Shah K. Smoking and passive smoking in cervical cancer risk: Pooled analysis of couples from the IARC multicentric case-control studies. Cancer Epidemiol Biomarkers Prev. (2011) 20:1379–90. doi: 10.1158/1055-9965.EPI-11-0284 21610224

[53] Zeng XT Xiong PA Wang F Li CY Yao J Guo Y. Passive smoking and cervical cancer risk: A meta-analysis based on 3,230 cases and 2,982 controls. Asian Pacific J Cancer Prev. (2012) 13:2687–93. doi: 10.7314/APJCP.2012.13.6.2687 22938442

[54] Gadducci A Barsotti C Cosio S Domenici L Riccardo Genazzani A. Smoking habit, immune suppression, oral contraceptive use, and hormone replacement therapy use and cervical carcinogenesis: A review of the literature. Gynecol Endocrinol. (2011) 27:597–604. doi: 10.3109/09513590.2011.558953 21438669

[55] Asthana S Busa V Labani S. Oral contraceptives use and risk of cervical cancer—A systematic review & meta-analysis. Eur J Obstet Gynecol Reprod Biol. (2020) 247:163–75. doi: 10.1016/j.ejogrb.2020.02.014 32114321

[56] Roura E Travier N Waterboer T de Sanjosé S Xavier Bosch F Pawlita M. The influence of hormonal factors on the risk of developing cervical cancer and pre-cancer: Results from the EPIC cohort. PloS One. (2016) 11:e0147029. doi: 10.1371/journal.pone.0147029 26808155 PMC4726518

[57] Peng Y Wang X Feng H Yan G. Is oral contraceptive use associated with an increased risk of cervical cancer? An evidence-based meta-analysis. J Obstet Gynaecol Res. (2017) 43:913–22. doi: 10.1111/jog.13291 28759170

[58] Li X Sundquist J Calling S Zöller B Sundquist K. Neighborhood deprivation and risk of cervical cancer morbidity and mortality: A multilevel analysis from Sweden. Gynecol Oncol. (2012) 127:283–9. doi: 10.1016/j.ygyno.2012.07.103 22819937

[59] McDonald YJ Goldberg DW Scarinci IC Castle PE Cuzick J Robertson M. Health service accessibility and risk in cervical cancer prevention: comparing rural versus nonrural residence in New Mexico. J Rural Heal. (2017) 33:382–92. doi: 10.1111/jrh.12202 PMC5939944 27557124

[60] Van Der Aa MA Siesling S Louwman MW Visser O Pukkala E Coebergh JWW. Geographical relationships between sociodemographic factors and incidence of cervical cancer in the Netherlands 1989-2003. Eur J Cancer Prev. (2008) 17:453–9. doi: 10.1097/CEJ.0b013e3282f75ed0 18714188

[61] Park MJ Park EC Choi KS Jun JK Lee HY. Sociodemographic gradients in breast and cervical cancer screening in Korea: The Korean National Cancer Screening Survey (KNCSS) 2005-2009. BMC Cancer. (2011) 11:257. doi: 10.1186/1471-2407-11-257 21682886 PMC3144456

[62] Gauri A Messiah SE Bouzoubaa LA Moore KJ Koru-Sengul T. Cervical cancer sociodemographic and diagnostic disparities in Florida: a population-based study (1981–2013) by stage at presentation. Ethn Heal. (2020) 25:995–1003. doi: 10.1080/13557858.2018.1471669 29732918

[63] Eggleston KS Coker AL Williams M Tortolero-Luna G Martin JB Tortolero SR. Cervical cancer survival by socioeconomic status race/ethnicity, and place of residence in Texas, 1995-2001. J Women’s Heal. (2006) 15:941–51. doi: 10.1089/jwh.2006.15.941 17087618

[64] Tiraki Z Yılmaz M. Cervical cancer knowledge, self-efficacy, and health literacy levels of married women. J Cancer Educ. (2018) 33:1270–8. doi: 10.1007/s13187-017-1242-3 28668992

[65] Wakwoya EB Gemechu KS Dasa TT. Knowledge of cervical cancer and associated factors among women attending public health facilities in eastern Ethiopia. Cancer Manag Res. (2020) 12:10103–11. doi: 10.2147/CMAR.S262314 PMC7569063 33116866

[66] Nang DW Tukirinawe H Okello M Tayebwa B Theophilus P Sikakulya FK. Prevalence of high-risk human papillomavirus infection and associated factors among women of reproductive age attending a rural teaching hospital in western Uganda. BMC Womens Health. (2023) 23:209. doi: 10.1186/s12905-023-02342-y 37118735 PMC10148521

[67] Prummel MV Young SW Candido E Nishri D Elit L Marrett LD. Cervical cancer incidence in Ontario women: Differing sociodemographic gradients by morphologic type (adenocarcinoma versus squamous cell). Int J Gynecol Cancer. (2014) 24:1341–6. doi: 10.1097/IGC.0000000000000217 25054446

[68] Parikh S Brennan P Boffetta P. Meta-analysis of social inequality and the risk of cervical cancer. Int J Cancer. (2003) 105:687–91. doi: 10.1002/ijc.11141 12740919

